# Mobile Mpox Vaccination in New York City Provided Flexible Community-Responsive Vaccine Access During the 2022 Global Mpox Emergency

**DOI:** 10.1093/ofid/ofaf053

**Published:** 2025-03-31

**Authors:** Joseph Osmundson, Julian L Watkins, Ashwin Vasan, Chris Hawke, Adam Baran, Jane R Zucker, Katya Murphy, Andrew Wallach, Theodore Long

**Affiliations:** Department of Biology, New York University, New York City, New York, USA; NYC Department of Health and Mental Hygiene, New York City, New York, USA; NYC Department of Health and Mental Hygiene, New York City, New York, USA; NYC Department of Health and Mental Hygiene, New York City, New York, USA; NYC Health and Hospitals (H+H), New York City, New York, USA; NYC Health and Hospitals (H+H), New York City, New York, USA; Department of Medicine, NYU Grossman School of Medicine, New York City, New York, USA; NYC Health and Hospitals (H+H), New York City, New York, USA; Department of Population Health, NYU Langone, New York City, New York, USA

**Keywords:** disease prevention, LGBTQ+ health, mpox, sexual health, vaccination

## Abstract

**Background:**

In May 2022, mpox (formerly monkeypox) began spreading globally through LGBTQ+ sexual networks. By August 2022, New York City (NYC) became the global epicenter of the mpox outbreak, with the highest number of cases reported in the United States. Here, we quantify the mpox vaccination effort, focusing on flexible and community-responsive mobile vaccination.

**Methods:**

We describe an on-site mpox vaccination strategy at commercial sex venues, nightlife venues, and pride and health centers, during August 1–November 15, 2022. Data were collected on doses, demographics, and event size to determine and evaluate vaccine uptake.

**Results:**

The on-site vaccination strategy resulted in 3358 JYNNEOS doses administered at 363 events at 58 locations, including 22 events at 2 commercial sex venues. Commercial sex venues in New York City closed at the height of the mpox epidemic. We show high uptake of the JYNNEOS vaccine at commercial sex venues, with as many as 60% of attendees of 1 event receiving a JYNNEOS vaccine dose on site. This was possible after New York City health agencies responded to community demand for second doses. Messaging about the importance and availability of vaccination at these parties was community-led. JYNNEOS vaccination via mobile clinics demonstrated less racial and geographic disparity compared with nonmobile vaccinations. We show no increase in mpox cases as commercial sex venues reopened with vaccination on site.

**Conclusions:**

These results demonstrate the success of a community-led rapid response to an emergent mpox outbreak, including at places where people meet for sex.

In 2022, New York City was the earliest and fastest growing epicenter for mpox cases in the United States [1], with nearly 3822 recorded cases in 2022 (Figure 1). After Pride celebrations in June, NYC's reported cases rose rapidly, reaching an average of >70 cases per day by August 1 (Figure 1). Further, although there was an available 2-dose vaccine, JYNNEOS (Modified Vaccinia Ankara vaccine, Bavarian Nordic), which is protective against infection [2], supplies were initially limited. Research from the United States [3] and New York State [1] demonstrates that 2 doses of the JYNNEOS vaccine are required for full efficacy, estimated to be between 66% and 88%, whereas 1 dose has an efficacy that varies between studies from 36% [3] to as high as 70% [4].

**Figure 1.**
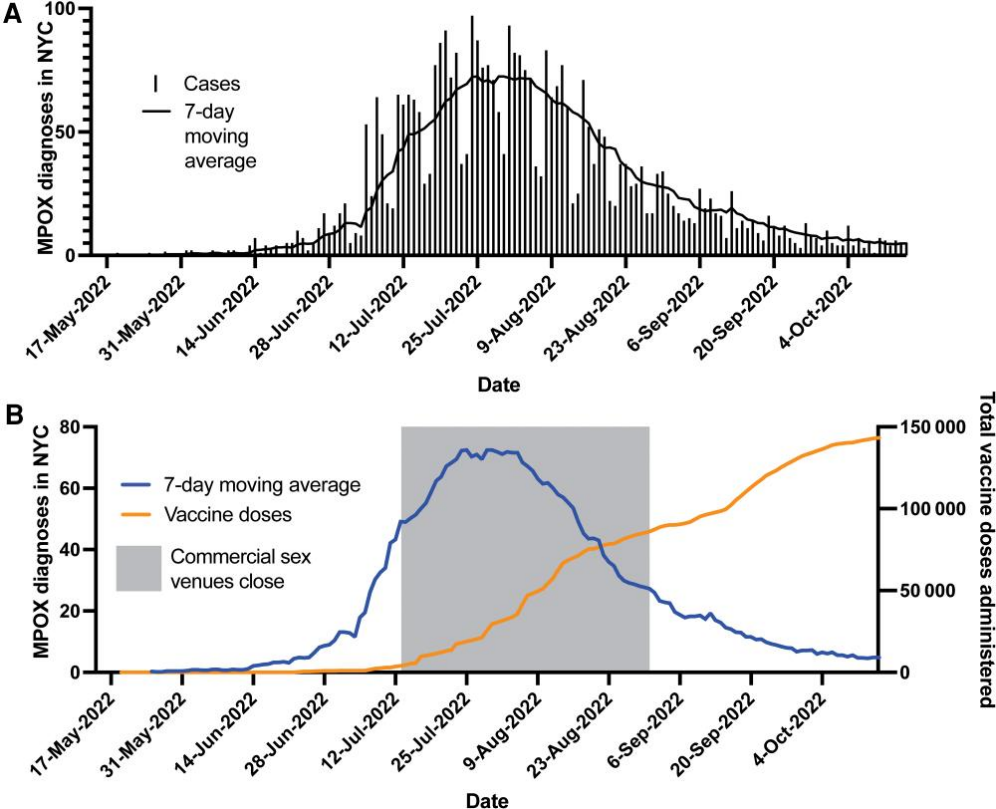
Mpox cases reported in NYC from May through October 2022. *A*, Mpox cases in NYC between May and mid-October (bars) and a 7-day rolling average (line). *B*, Behavioral changes (gray box indicates closure of commercial sex venues) and JYNNEOS vaccinations (right *y*-axis) correspond with a decrease in the new mpox cases in NYC. Abbreviation: NYC, New York City.

The global mpox outbreak showed a tight association with LGBTQ+ sexual networks, especially but not only in men who have sex with men (MSM) [5]. Early spread of mpox in Europe was linked to large venues where LGBTQ+ networks meet, including circuit parties and saunas [6]. Later data from the Centers for Disease Control and Prevention (CDC) suggest significant behavioral changes within LGBTQ+ sexual networks in response to mpox [7].

Prevention of mpox infection can be achieved through vaccination and/or behavioral changes, particularly involving sex and other forms of intimate contact [7, 8]. Modeling shows that high levels of immunity in a small number of people, if those people are very highly connected in a sexual network, can have outsized effects in lowering the reproduction number, or number of cases stemming from each case, in a population of both susceptible and nonsusceptible individuals, and therefore provide protection to the community at large [9]. Bacterial sexually transmitted infections (STIs) show similar patterns; in a large study of HIV preexposure prophylaxis (PrEP) efficacy that also examined bacterial STIs, 86% of the infections were connected to 39% of the participants, whereas 40% of participants did not have a bacterial STI during the study [10]. Targeting interventions to those individuals, particularly in an emergency with vaccine scarcity, was therefore a goal of both community advocates and city officials.

NYC has a long history of public health interventions at LGBTQ+ venues, including for HIV testing, STI screening, and meningitis vaccination [11, 12]. Our team, including independent researchers and community leaders, worked to establish trusted connections between city health officials and the owners and operators of LGBTQ+ nightlife and commercial sex venues such that vaccination could be provided on site. Here we describe an mpox vaccination pop-up program using a collective impact approach [13, 14]. This approach to community collaboration is a model for tailored engagement that meets the diverse needs of marginalized communities during a public health emergency response in limited-resource settings and beyond.

## METHODS

### On-site Vaccination as Part of a Vaccine Distribution Strategy

The NYC Department of Health and Mental Hygiene (DOHMH) is the city's public health authority; in response to public health emergencies, the DOHMH is responsible for disease surveillance and epidemiology, clinical guidance, and mass vaccination.

The DOHMH oversaw the administration of >150 000 doses of JYNNEOS during the mpox response during June–October (Figure 1) through (1) mass vaccination sites, (2) vaccine administration sites at DOHMH sexual health clinics, and, (3) as vaccine supply improved, distribution of vaccine directly to medical facilities. Vaccine eligibility was not restricted by jurisdiction of residence, but some providers and mobile events may have requested an NYC address when registering clients.

NYC Health + Hospitals (NYC H + H) is a public/private partnership with 11 hospitals and >50 community health centers. As part of its coronavirus disease 2019 (COVID-19) response, NYC H + H built a mobile testing, vaccination, and treatment platform using vans to reach underserved communities and large events. This preexisting platform became the basis for mpox mobile vaccination.

### Identification and Classification of On-site Vaccination Locations

In partnership with community members, we identified 58 locations in NYC for on-site vaccination. Our authors include community experts and event hosts who participated in this process.

For analysis, we classified locations into 3 categories: commercial sex venues, bars/nightlife establishments, and pride and health centers. At commercial sex venues, attendees remove most or all clothes at the entrance and the majority participate in some sexual activity; these activities were reported by event hosts and volunteer mpox outreach workers on site. Nightlife included all bars and events where alcohol is served. Pride centers were LGBTQ+-focused community venues without regular health care on site. Health centers were LGBTQ+-oriented sites where other health care, such as STI and HIV testing, is also available, but mpox vaccination services were not being otherwise offered during the study period. We combine pride and health centers because they are mixed-use affinity spaces open during the day where individuals can socialize and access services.

We grouped all nightlife together, including bars with and without designated areas for sex (according to attendees and hosts), because a survey during our study period showed that people in LGBTQ+ sexual networks had sexual and/or close physical contact at a wide variety of bars regardless of whether they have a dedicated space for sexual contact (Keletso Makofane, personal communication) [15].

NYC has several commercial sex venues; of these, 2 large spaces regularly host parties for LGBTQ+ people. These venues, including one in midtown Manhattan and another in Brooklyn, typically host 2–4 large events (>100 participants) a week. Initial contact with these venues was made by community members with trust and knowledge of these events who facilitated ongoing communication between the DOHMH, H + H, community leaders, and event hosts.

We describe 2 parties as case studies, 1 party for gay cisgender men aged 18–40 (Party 1) and another monthly party that is trans-inclusive and gender diverse (Party 2). Party 1 meets weekly, alternating between Manhattan and Brooklyn locations. Because these recurring events often include similar participants, we grouped the events by organizer and use “Party 1” to refer to all events associated with a particular organizer throughout the study period.

### On-site Planning

Mpox vaccine vans, each able to administer 60 doses of JYNNEOS per event, were parked outside of LGBTQ+ health and pride centers, nightlife venues, and commercial sex venues (Supplementary Figure 1). For events with >60 expected doses based on host feedback, >1 van was provided.

At commercial sex venues, vans were positioned near but not directly outside the venues in order to both ensure anonymity for those getting vaccinated and to protect the owners and operators of the venues from scrutiny. Locations were previously agreed upon. Unlike COVID-19 vaccine vans in New York, mpox vaccine vans were unmarked white vehicles that supported staff inside a venue or under a blue tent on the sidewalk to administer vaccine (Supplementary Figure 1). When possible, a volunteer liaison was at the entrance to the event answering questions and providing information and the location of the van. Because of the rapidly shifting nature of the emergency, the liaison was a community-based volunteer in close contact with city health officials. When not possible, we worked with the event staff to have them remind people about the vaccine vans as they entered and left the venue. Reminders were also sent by party promoters in their regular text, social, and email advertisements for the events, with language coordinated through the community volunteers. Event organizers emphasized the availability of mpox vaccines on site in top-line messaging, including Party 2 naming their event “Sub/Cute/Anus,” a play on the availability of subcutaneous vaccinations, whereas other sites prioritized intradermal. Event organizers emphasized the availability of second JYNNEOS doses.

JYNNEOS vaccination via mobile clinics began at pride and health centers on 8/01/2022, at nightlife venues and bars on 8/16/2022, and at commercial sex venues on 9/03/2022. Locations were selected based on interest in on-site vaccination, the size of the event, the sexual behaviors that occur on site and the likelihood of mpox transmission, and the ability to reach underserved communities. While Jynneos vaccination shifted to an intradermal dosing strategy in August 2022, requirements for training and oversight at the mobile units led to ongoing subcutaneous vaccination. Community volunteers communicated the availability of subcutaneous doses regardless of preexisting condition to event promoters at commercial sex venues.

The monitoring period was performed on site in the tent set up by the mobile unit (Supplementary Figure 1). Individuals were informed that the vaccination process would take ∼30 minutes, including intake and the monitoring period. Outreach volunteers noted hours of operation such that individuals who did not want to wait 30 minutes before entering the event could instead receive vaccination as they left the event.

### Descriptive Analysis

Data on dose type, location, and the demographics of the recipient of mobile vaccinations were provided by the vendor, allowing for real-time data analysis and communication with community stakeholders. Comparison data from citywide vaccination (eg, nonmobile sites) were either publicly available or provided by the DOHMH from the New York Citywide Immunization Registry (CIR). For mobile vendors, vaccinations were reported to the CIR using the zip code of the vendor, not the location of the site where the vaccination was administered. Data about site location for doses administered by the mobile vans were collected directly by vendors. Due to the differences in data sources used (data collected by the vendor vs vaccination data reported to the CIR), direct comparisons between mobile and nonmobile vaccination cannot be made. We therefore provide a descriptive analysis. The data for Latinx/Hispanic race/ethnicity are difficult to compare directly due to differences in reporting between the mobile vendor and the data obtained via the CIR (eg, at mobile clinics, a Latinx person could select White, Black, or other for their race and then Latino/Hispanic for their ethnicity, vs having Hispanic included directly in the options for race).

The total number of weekly doses, dose type, and event were calculated in R and Python and plotted in Prism. Information about individual events, including demographics and flyers, were provided by event hosts. Event size was provided by the event hosts, whose staff gave a unique number to each participant and recorded that number in a database.

### Mapping Studies

The zip code of vaccination site and zip code of residence of those vaccinated were mapped in Python using geopandas and matplotlib. Analysis of citywide doses in this study was restricted to NYC residents.

## RESULTS

During August 1, 2022, through November 15, 2022, mobile vans administered 3358 JYNNEOS doses during 363 events at 58 locations: 2495 JYNNEOS doses at 298 events at community and pride centers, 433 doses at 42 events at nightlife venues, and 430 doses at 23 events at commercial sex venues. Nearly all doses administered at mobile units during the study period were administered to NYC residents (96%). A total of 117 113 JYNNEOS doses were administered citywide to NYC residents and reported to the DOHMH during this time period.

### Case Studies

**Figure 2.**
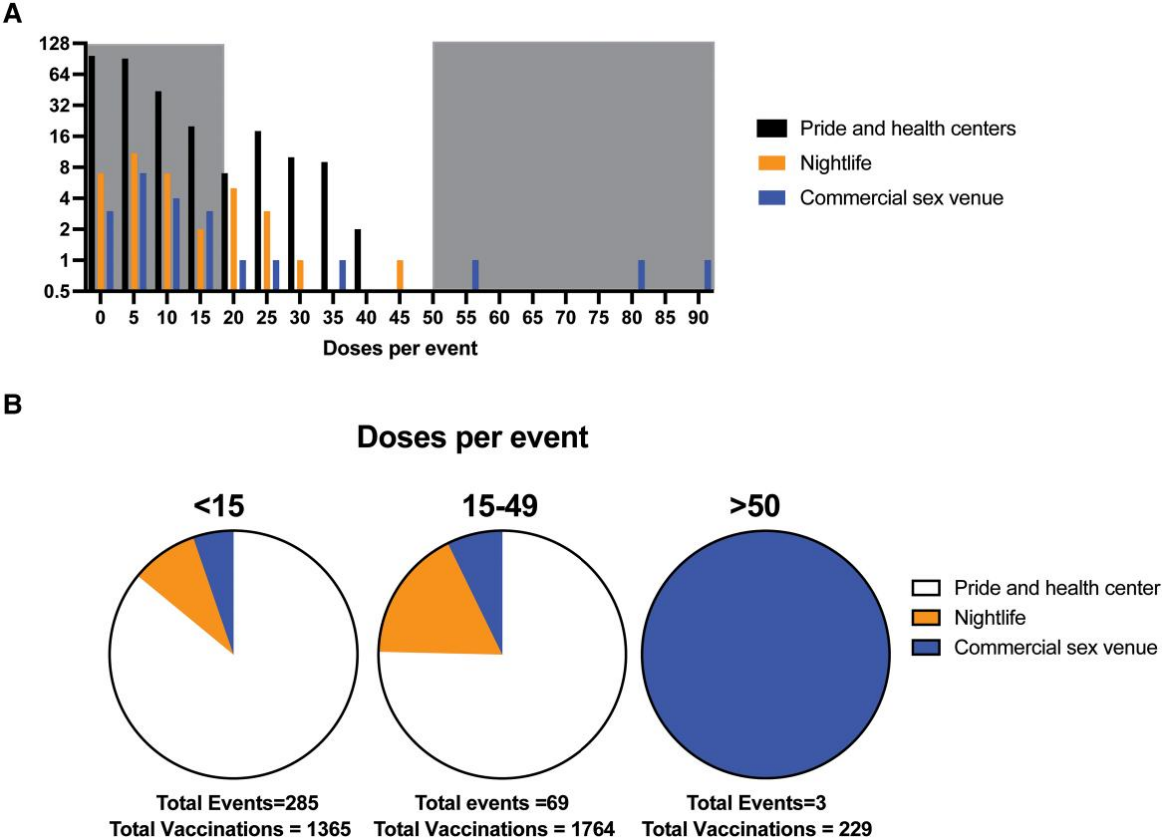
Mobile clinics at commercial sex venues, but not at nightlife venues or pride centers, administered >50 JYNNEOS doses at single events. *A*, Histogram plotting the number of doses per event. *B*, Events were categorized as high-vaccination events (>50 doses), medium-vaccination events (15–49 doses), or low-vaccination events (<15 doses) and visualized by pie charts. The majority of doses were administered at medium-vaccination events (n = 1764).

To evaluate the differences between mobile vaccination sites at different types of events, we plotted a histogram of the number of doses administered per event. The only 3 events that vaccinated >50 individuals were located at commercial sex venues (Figure 2). All 3 events occurred during September 10, 2022, through September 17, 2022.

Commercial sex venues in NYC voluntarily closed in mid-July in response to the mpox outbreak. On September 3, given increased vaccination levels and lower mpox levels, commercial venues reopened in NYC (Figure 1*B*). Outreach before September 3 facilitated mobile vaccine units on site from their reopening.

When Party 1 reopened, mobile clinics offered only first doses of JYNNEOS, vaccinating 13 individuals. A researcher on site spoke to event attendees, many of whom reported that they would have received a vaccine if second doses were offered.

After a feedback meeting with the DOHMH, H + H, and community researchers on September 6, 2022, city officials allowed the administration of second doses at commercial sex venues for anyone vaccinated 28 days prior, citing increasing supply and the need for robust mpox protection for individuals who participate in group sex [9]. Individuals who attend commercial sex venues tend to have a higher number of sexual partners [16, 17], and mpox spread was previously linked to commercial sex venues [6]. Second doses elsewhere in NYC were then available only for those who received the first dose more than weeks 12 prior.

**Figure 3.**
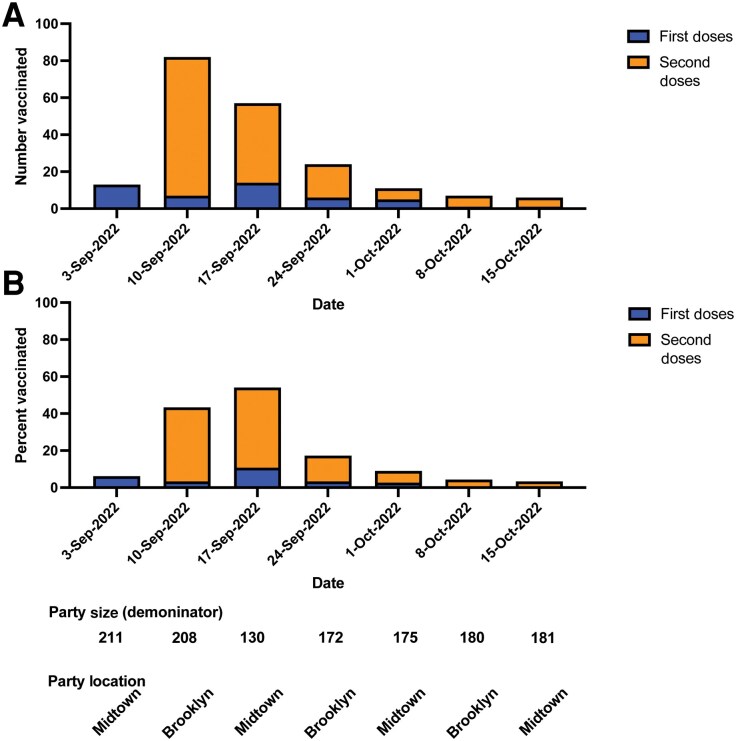
Vaccinations at Party 1 increased rapidly for 2 weeks following the availability of second JYNNEOS doses on site. First and second doses administered were reported by the vendor, and the denominators for party size were reported by the party hosts. *A*, Number of vaccinations. *B*, The percentage each event vaccinated on site.

On September 10, Party 1 was held with 208 people attending, and 82 vaccine doses (75 second doses) were administered on site, representing 39% of event participants (Figure 3). Party 1 organizers had sent messaging to participants informing them that second mpox vaccine doses would be available on site.

This pattern held for the subsequent week (September 17) at Party 1 in Manhattan. At a party of 130 people, 57 doses, including 43 second doses, were administered (Figure 3).

Party 2, a monthly trans-inclusive event, was also held September 17. The host named their event that evening “Sub/Cute/Anus” as a play on subcutaneous vaccine administration. At an event of 151 people, 90 vaccine doses (74 second doses) were administered, with nearly 60% of the participants receiving a dose of JYNNEOS vaccine on site.

We closely monitored case data in the month after commercial sex venues reopened to identify a rebound of mpox transmission; if case numbers rebounded, event hosts would again pause their parties. Even as sex venues reopened with vaccinations on site, mpox cases in New York City continued to fall (Figure 1). On September 16, 2022, the DOHMH opened appointments citywide for second dose vaccine administration for anyone who received their initial dose at least 28 days prior.

### Commercial Sex Venues Administered a Higher Proportion of Second Doses Than Other Sites

**Figure 4.**
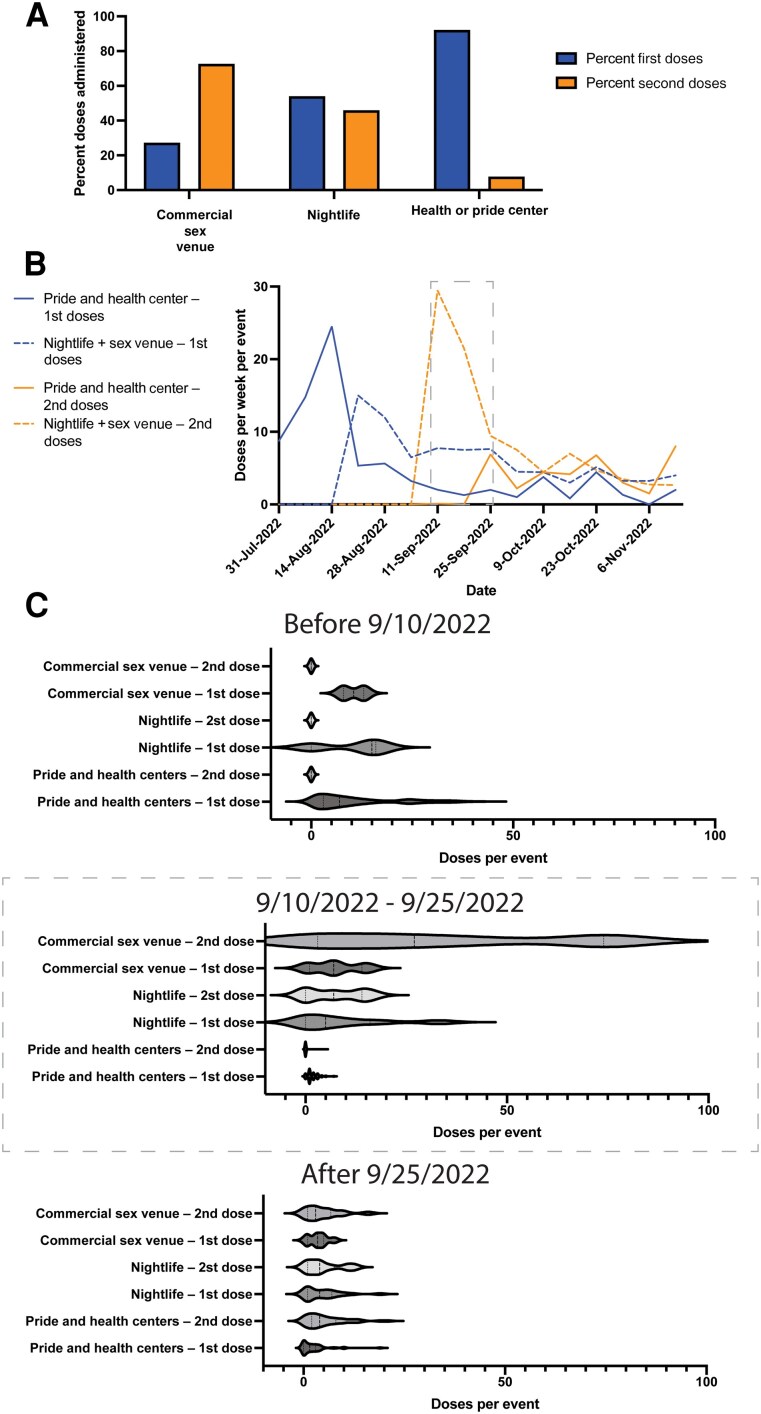
A small number of events at commercial sex venues administered a large proportion of the second JYNNEOS doses citywide. *A*, The percentage of first and second doses administered at commercial sex venues, nightlife venues, and pride and health centers. *B*, The number of first and second doses administered weekly by dose (first and second) and event type (pride and health center and nightlife/commercial sex venue). *C*, Violin plots showing all individual events with mobile JYNNEOS vaccine units at pride and health centers, nightlife events, and commercial sex venues by dose type and time frame, including all doses before 9/10/2022 (top panel), doses between 9/10/2022 and 9/25/2022 (middle panel), and doses after 9/25/2022, when second JYNNEOS doses were more available citywide (bottom panel).

Because commercial sex venues administered a high proportion of second JYNNEOS doses (Figure 3), we plotted the proportion of first and second doses by mobile event type (Figure 4*A*). We found that events at commercial sex venues administered 71.4% of second doses compared with only 11.4% at pride and health centers (Figure 4*A*). A total of 799 second JYNNEOS doses were administered by mobile clinics, with commercial sex venues accounting for 307 doses, nightlife 206 doses, and pride and health centers 286 doses.

This high proportion of second doses at commercial sex venues could be explained by the timing of availability of doses, the different populations, and/or the administration of subcutaneous as opposed to intradermal doses, which were not widely available at any other location in NYC except for medical exemptions. We plotted first and second doses administered at events by category over time. We combined data from commercial sex venues and nightlife for clarity and because sexual contact occurs at both type of events (Figure 4*B*). Mobile clinics began vaccinations at pride and health centers on August 1, 2022, when vaccine scarcity was high and only first doses were being administered. On August 15, doses were offered outside nightlife venues, with another peak of initial uptake (Figure 4*B*).

When commercial sex venues reopened with mobile clinics on site and second JYNNEOS doses available, we again saw a surge in uptake (Figure 4*B*). Because vaccinations were available via mobile clinics at commercial sex venues but not yet widely available elsewhere from 9/10 to 9/25/2022, we disaggregated data from this period, highlighting the 3 events with >50 doses administered (Figure 4*C*).

### Demographics of JYNNEOS Vaccinations at Mobile Clinics

**Figure 5.**
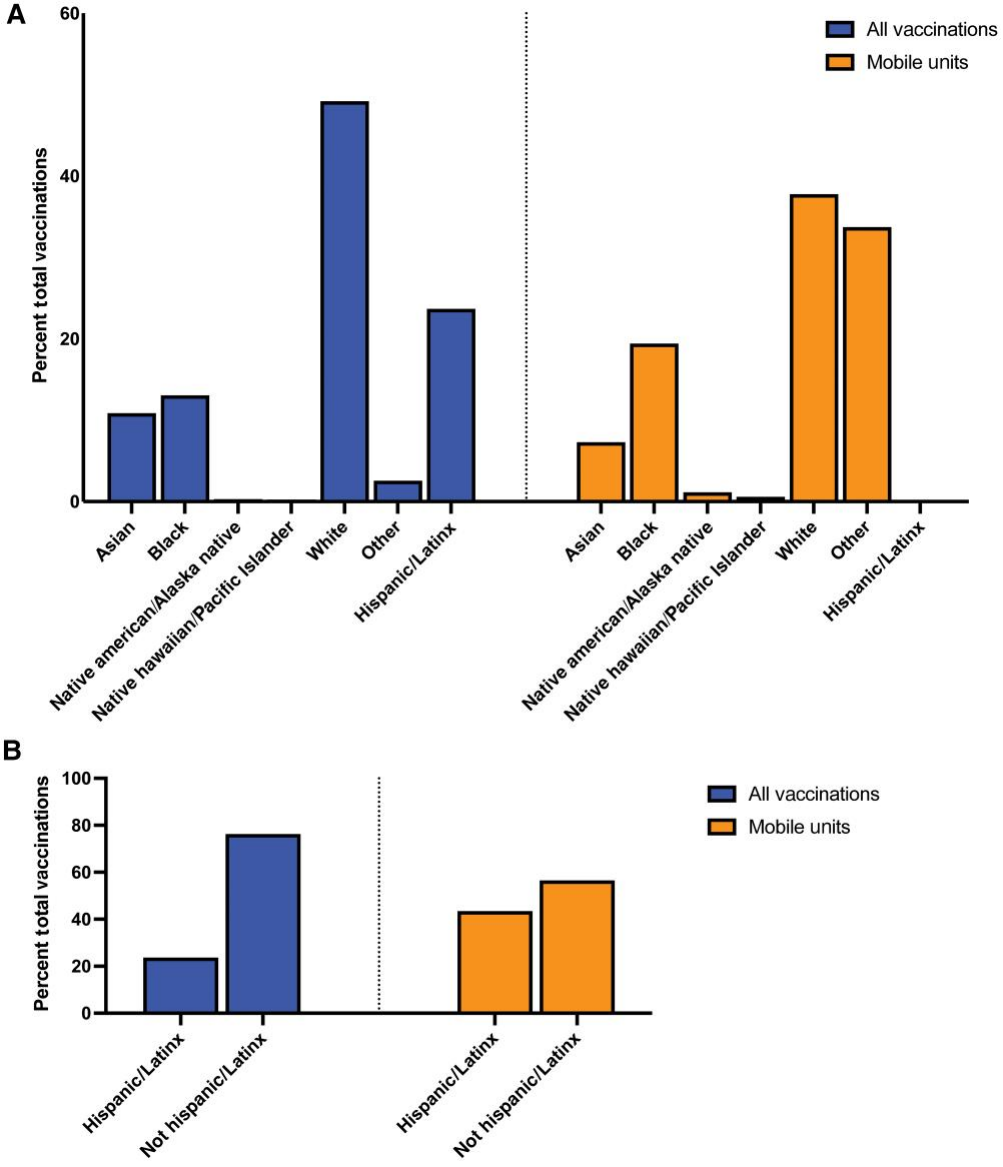
Compared with all JYNNEOS doses administered in NYC, mobile vaccination units served a larger proportion of non-White residents. *A*, The racial demographics by percentage of vaccine recipients as reported to the CIR are plotted for all NYC doses in 2022 (left panel), and the racial demographics by percent vaccine recipients as reported by the vendor at NYC's mobile clinics (right panel). *B*, The percentage of all NYC vaccine recipients who self-reported Hispanic/Latinx identity via the CIR (left panel) and NYC's mobile clinics as reported by the vendor (right panel). Abbreviations: CIR, New York Citywide Immunization Registry; NYC, New York City.

Racial disparities in citywide JYNNEOS vaccination remained throughout the study period (from August 1 through November 15), with half of those receiving doses citywide being White (50.1%, Figure 5); only 12.7% were Black. At mobile clinics, 19.4% and 43.5% of vaccine recipients were Black and Latinx/Hispanic, respectively (Figure 5*A*; Supplementary Table 1). During the study period citywide, 22.3% of vaccine doses were administered to people who identified as Latinx/Hispanic (Figure 5*B*).

Geographic disparities in mpox vaccinations in NYC persisted throughout the study period. We compared the zip codes of individuals receiving the JYNNEOS vaccine at mobile clinics vs all sites. While nonmobile vaccination citywide prioritized residents of zip codes in west Manhattan and central Brooklyn (n = 117 113), the smaller number of mobile doses (n = 3358) were more evenly distributed across NYC zip codes (Supplementary Figure 3).

## DISCUSSION

We demonstrate the importance of offering JYNNEOS vaccination on site at commercial sex venues during the 2022 global mpox outbreak in NYC (Figure 3), with as many as 60% of attendees at 1 event receiving a dose. Additionally, the reopening of commercial sex venues and events was not associated with a subsequent increase in mpox cases; while sporadic cases of mpox continued in NYC, weekly cases declined through our study period and never rose above 10 per week through the end of 2023, compared with a peak of >500 in mid-July 2022. Therefore, we report an example of sustained outbreak control in the post–emergency response period where behaviors associated with increased transmission began to return to a pre-outbreak baseline [7] without a rebound of new infections.

The JYNNEOS vaccine is authorized as a 2-dose series given at least 4 weeks apart [2]. The proportion of second JYNNEOS doses was higher at commercial sex venues than other events with mobile vaccination (Figure 4). This high uptake of second JYNNEOS doses at commercial sex venues could be due to the timing of mobile vaccination, when vaccine scarcity remained, the high vaccine acceptability among the population that attends commercial sex venues, the availability of subcutaneous doses whereas other vaccination sites in NYC administered via the dose-sparing intradermal method, wide community recognition of the emergent public health threat, and/or knowledge that those with a higher number of sex partners are at greater risk for mpox. Citywide administration of second JYNNEOS doses remained <50 000 during the study period compared to nearly 100 000 first doses (Supplementary Figure 2).

Additionally, we show that when mobile clinics offer vaccinations at new sites, an increase in vaccinations occurs (Figure 4*B*), indicating that ongoing community partnerships and new mobile vaccination locations may continue to address populations without previous access to vaccination in the context of an emergency response. As shown previously in other contexts [8, 18], we demonstrate that mpox cases slowed and declined in NYC before July 25 (Figure 1*B*), when only 18 073 JYNNEOS vaccinations were administered citywide, representing only 16.3% of the eligible population determined by the CDC [19].

We found anecdotally that some events with high numbers of doses administered advertised with flyers, texts, and emails made by the hosts in their own language and design (Figure 4); previous work in NYC includes outreach materials developed by city officials [11, 12].

One limitation of our ability to draw robust correlations is that, due to the emergency nature of this response, we did not collect data on various types of outreach at all sites, and therefore cannot empirically determine differences in approaches. We also did not study whether the ongoing availability of subcutaneous doses at mobile clinics, as opposed to dose-sparing intradermal administration, impacted uptake of vaccination.

Another limitation of our study includes that only demographic data were collected at the time of vaccination and comparison data were available only via the CIR. While other research demonstrates broad behavioral changes in LGBTQ+ sexual networks to avoid mpox exposure, we cannot address whether individuals who attended commercial sex venues altered their sexual practices during the period when commercial sex venues were closed. Additional study of the attitudes toward first- and second-dose vaccination at different types of venues would be useful to understand the reasons behind the seemingly disproportionate uptake of second doses at commercial sex venues. These results could inform not just ongoing mpox vaccination, but the use of mobile clinics for other types of sexual health care, including HIV testing, HIV PrEP education, and doxycycline postexposure prophylaxis to prevent bacterial STIs.

Because commercial sex venues self-select for those highly connected in LGBTQ+ sexual networks [16, 17, 20, 21], interventions and care offered on site could have ripple effects on transmissible infectious diseases to others beyond those who directly attended the event [9]. Rates of bacterial STIs, including chlamydia, gonorrhea, and syphilis, continue to rise both in NYC [22] and nationwide [23]. We demonstrate high uptake of sexual health care at commercial sex venues in the context of an outbreak. Outreach and mobile care at sites where people gather, including where they gather specifically for sex, should become an ongoing tool to reverse this unyielding trend. Stigma and shame are barriers to sexual health care [24] and may contribute to increases in anal sex without condoms [25]. Providing affirming and culturally sensitive sexual health care where people meet for group sex remains an underused tool in NYC.

While some nightlife and commercial sex venue events administered fewer total doses, targeted mobile clinics in underserved zip codes or for events with communities disproportionally impacted by mpox remained a vital part of NYC's emergency response.

Sexual health care in NYC, like elsewhere, was disrupted during the COVID-19 pandemic [26]. The successful JYNNEOS vaccination efforts at nightlife events and commercial sex venues required reliance on both existing and new relationships with LGBTQ+ communities and trust building, even during the height of the mpox outbreak. Community members held leadership positions in this project from its inception, through the design of outreach materials and the production of this manuscript. Maintaining these relationships beyond the initial crisis will be critical to further assist with much-needed outreach for other diseases, such as HIV and other STIs, and in response to other new emerging infectious diseases and STIs.

Our results highlight the importance of an intentional, equity-centered approach that prioritizes partnership with community groups to leverage their expertise to address barriers to care faced by the most marginalized members of impacted communities [13, 14]. This work would not be possible without the emergency public health infrastructure created in response to the COVID-19 pandemic [13]. Cost remains a major barrier to doing this work routinely. Prioritization of quantity, for example, rapid administration of a number of vaccinations, often takes precedence over the quality and sustainability of tailored engagement (fewer vaccinations at sites to reach undervaccinated communities) and the impact interventions may have on marginalized communities [14]. To truly be response-ready and address ongoing disparities in sexual health care, jurisdictions will need dedicated investment in tailored community engagement to build trust and maintain the infrastructure to support community-centered efforts to address threats to public health.

## Supplementary Material

ofaf053_Supplementary_Data

## Supplementary Data

Supplementary materials are available at *Open Forum Infectious Diseases* online. Consisting of data provided by the authors to benefit the reader, the posted materials are not copyedited and are the sole responsibility of the authors, so questions or comments should be addressed to the corresponding author.
