# Supplementary figures and images for: Mobile Mpox Vaccination in New York City Provided Flexible Community-Responsive Vaccine Access During the 2022 Global Mpox Emergency
Source: Open Forum Infect Dis. 2025 Mar 31;12(4):ofaf053. doi: 10.1093/ofid/ofaf053 (PMC11949096; doi:10.1093/ofid/ofaf053)

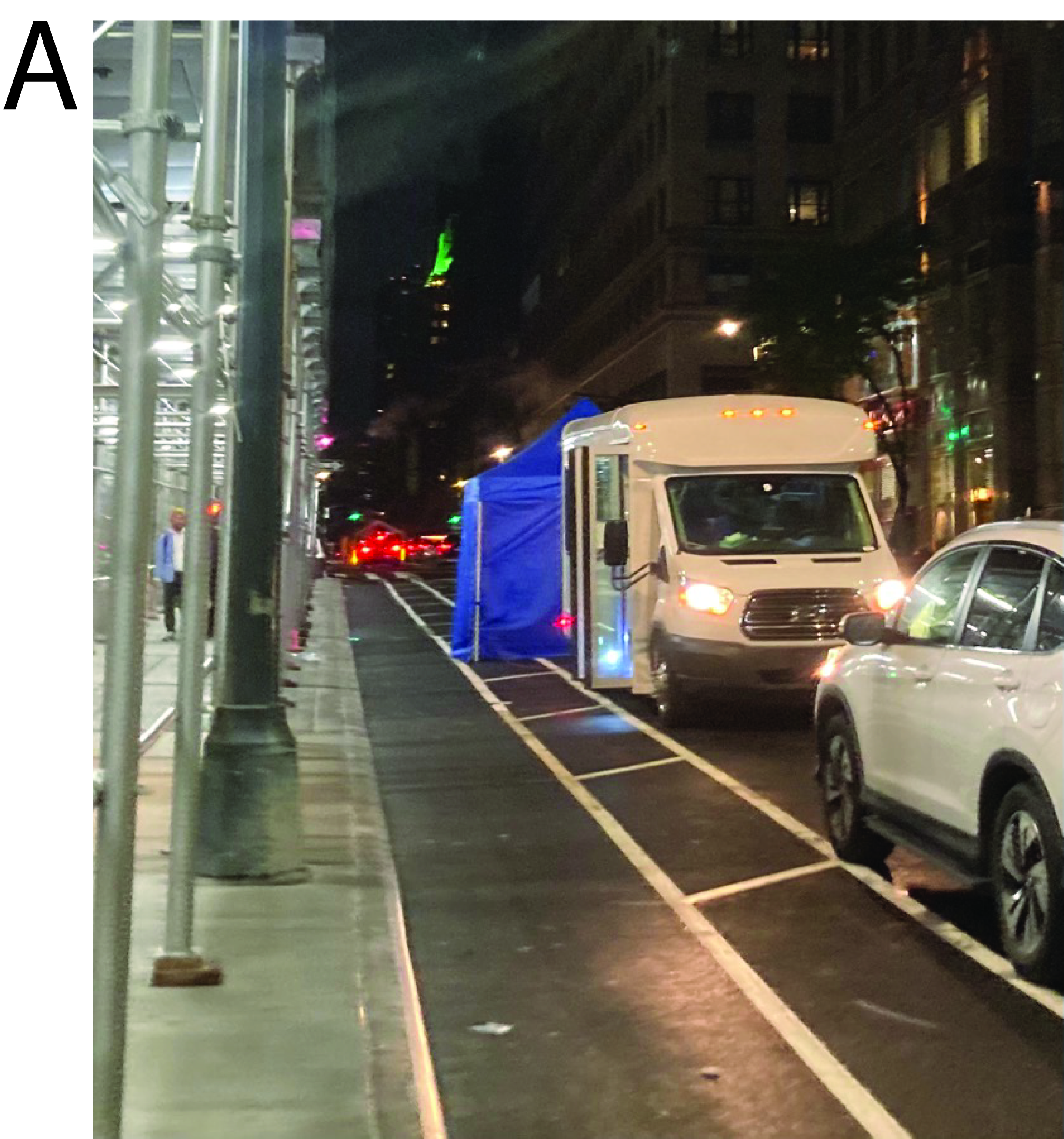

Supplement: ofaf053_Supplementary_Data [file ofaf053_supplementary_data.zip › FigureS1-new.tif]

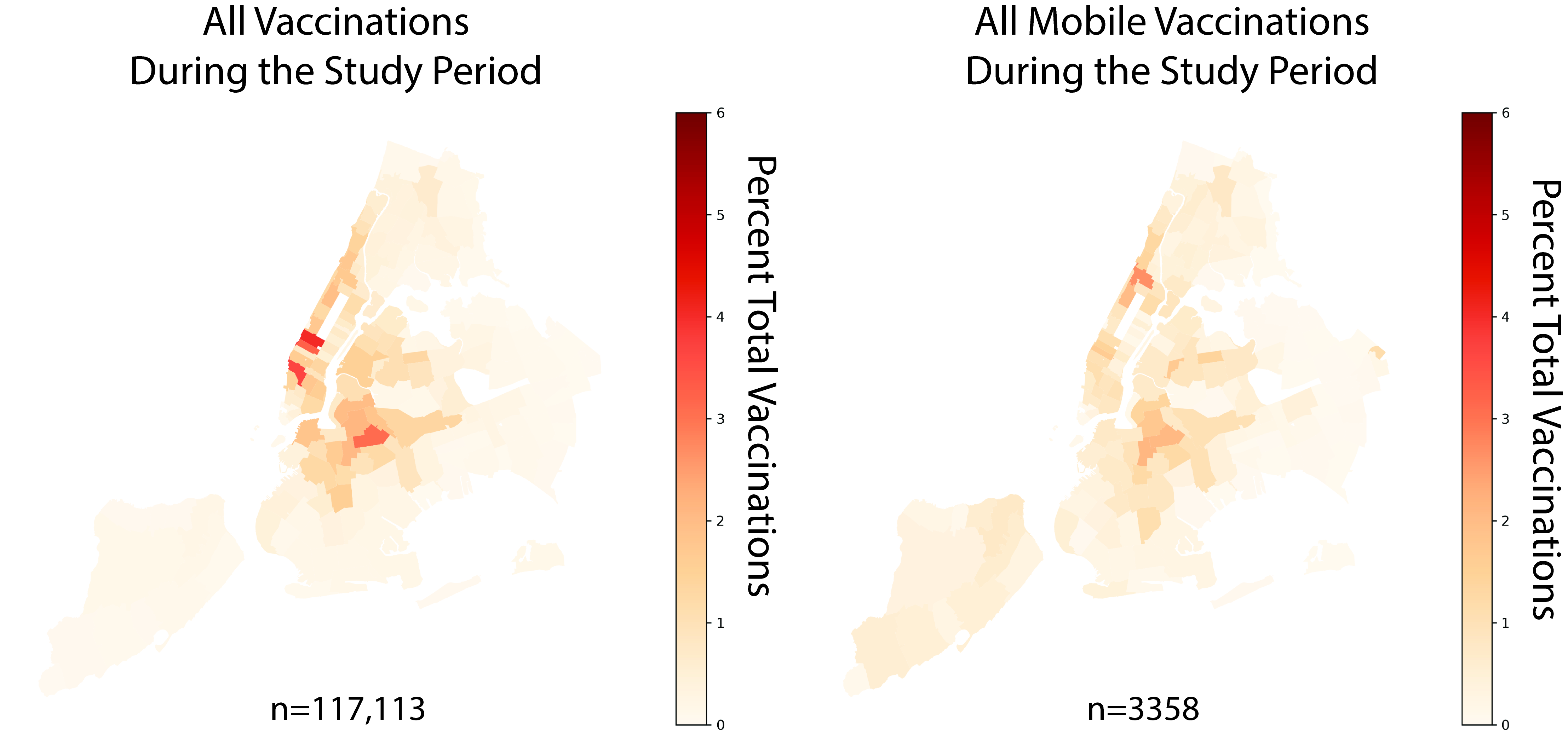

Supplement: ofaf053_Supplementary_Data [file ofaf053_supplementary_data.zip › Osmundson-FigureS2.tif]

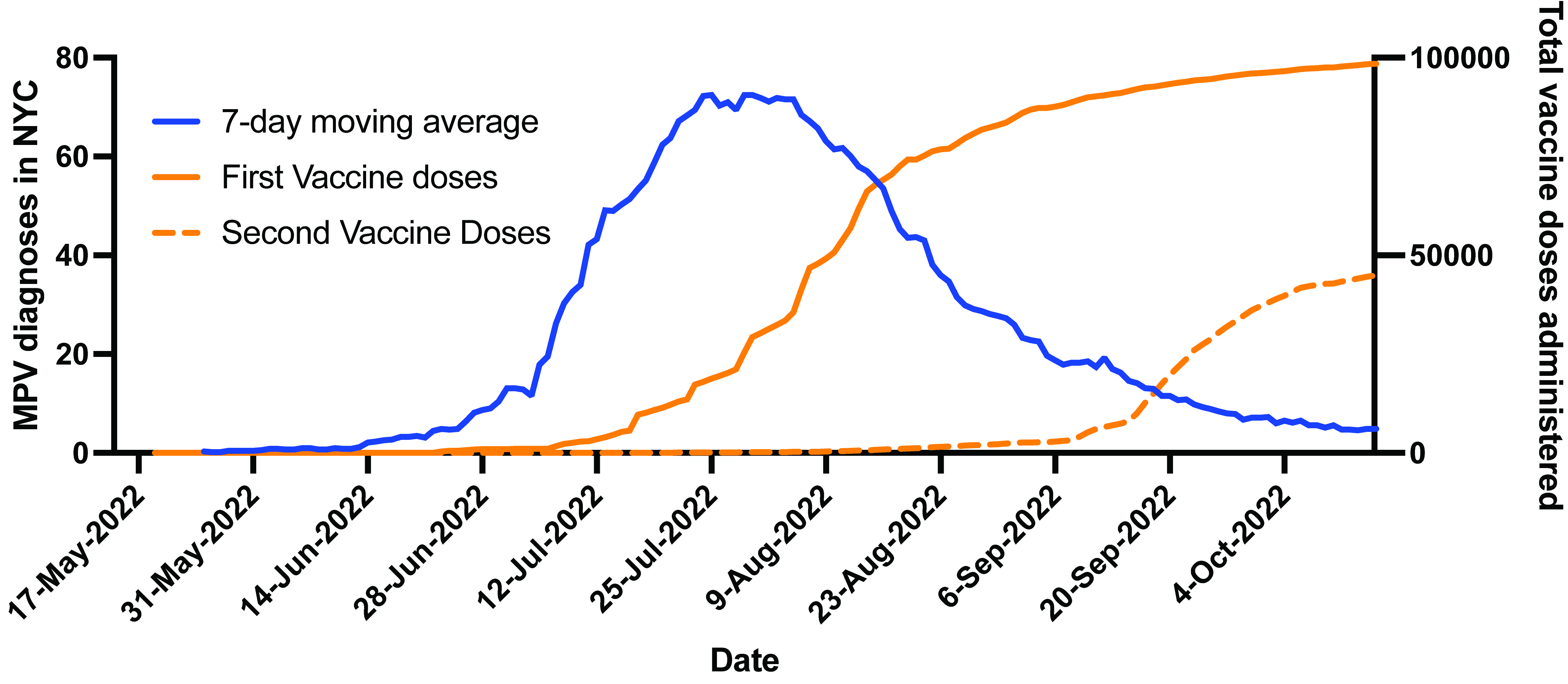

Supplement: ofaf053_Supplementary_Data [file ofaf053_supplementary_data.zip › Osmundson-FigureS3.tif]
